# Cultural moderation in sports impact: exploring sports-induced effects on educational progress, cognitive focus, and social development in Chinese higher education

**DOI:** 10.1186/s40359-024-01584-1

**Published:** 2024-02-22

**Authors:** Qinglei Wang, Nor Eeza Zainal Abidin, Mohd Salleh Aman, Nina Wang, Luhong Ma, Pan Liu

**Affiliations:** 1 Faculty of Sports and Exercise Science, Universiti Malaya, Kuala Lumpur, Malaysia; 2 Department of Educational Psychology & Counselling, Faculty of Education, Universiti Malaya, Kuala Lumpur, Malaysia; 3 Department of Sport Studies, Faculty of Educational Studies, Universiti Putra Malaysia, 43300 Seri Kembangan, Selangor Malaysia

**Keywords:** Perceived Educational progress, Sports activities, Sports-induced cognitive focus, Psychological well-being, Cultural Context

## Abstract

**Background:**

This research examines the nuanced challenges confronting Chinese university students within the dynamic milieu of Chinese education. The study comprehensively investigates factors encompassing educational progress, social development, cognitive focus, and Psychological Well-being (PWB), specifically emphasizing the role of sports participation.

**Methods:**

To scrutinize the moderation-mediation nexus between cultural context and social development, a distribution of 500 questionnaires was administered to Chinese university students, yielding 413 responses, corresponding to an 82.6% response rate. Methodologically, this study employed moderation and mediation analyses, incorporating statistical techniques such as a principal component matrix, factor analysis, and hierarchical regression.

**Findings:**

Prominent findings underscore the significant impact of age on educational progress, shaping the trajectory of academic advancement. Cumulative Grade Point Average (CGPA) emerges as a promising metric, establishing a link between academic performance and educational progress. Active involvement in sports and physical activities (PSPA) positively affects academic performance and study habits. Participation in sports teams and clubs (ISTC) enriches social development by nurturing interpersonal relationships, teamwork, and leadership skills. Sports activities (ESA) correlate with enhanced cognitive focus and improved psychological well-being. Significantly, the findings unveil a nuanced association between Perceived Social Development Through Sports (PSDTS) and educational progress.

**Conclusions:**

Cultural Context (CC) moderates PSDTS, Sport-induced Cognitive Focus (SICF), and PWB, influencing educational progress. This study emphasizes the need for enhanced support systems—academic guidance, awareness, sports programs, and cultural competence training—to advance student well-being and academic achievement in China, fostering an empowering educational environment for societal progress.

## Introduction

A discernible nexus between personal well-being and cognitive performance has been established in the present epoch. Research underscores the centrality of individual development, encompassing academic progress and mental health [1]. Sports and physical activities are recognized as potent facilitators of human development [2]. This study investigates the ramifications of sports on Chinese university students, specifically focusing on academic achievements, social maturation, mindfulness, and psychological well-being. By examining these interrelated dimensions, the research seeks to elucidate the nuanced contributions of sports to the holistic well-being of Chinese tertiary education attendees.The development of physical education curricula in Chinese universities is summarised in Table 1, highlighting essential policy changes, educational goals, and syllabi requirements.

**Table 1:** Chinese higher education’s evolutionary policies regarding physical education (1979–2014)

Year	Policy/Document	Focus	Teaching Goals	Curriculum Guidelines	Syllabus Requirements	Student Involvement
1979	Yangzhou Conference	Mastery of sports techniques; Strengthening physical fitness	Realizing physical fitness of teachers and students	Physical Education (PE) Curriculum: 1st and 2nd-grade courses, 140 h	Mandatory for 1st and 2nd-grade students	-----
1992	PE Curriculum Guiding Outline	Awareness of physical education; Enjoyment of physical ability; Ideological education	People-oriented concept; Students’ needs	-----	-----	-----
2002	Revised PE Curriculum	Sports participation, skills, physical and mental health, social adaptation	Move from three to five dimensions	Freshman and sophomore courses (144 h); Optional for others	On/Off-campus; ~30 students per class	Mandatory for freshmen and sophomores; Elective for others; Counts into credits
2014	Basic Standards for PE	Mandatory for freshmen and sophomores; Elective for juniors, seniors, and graduates	-----	-----	Maximum 30 students per class	-----

Source: Adapted from Wei & Wang [3]

The research employs a theoretical framework that draws on various critical theories to comprehensively investigate the intricate relationship between sports involvement and academic progress in the specific context of China. The variable Perceived Enhancement in Educational Progress (PEP) is grounded in the ‘Social Cognitive Theory,’ positing that individuals acquire knowledge and skills through observation and reciprocal interactions. The PEP program considers academic performance and study habits, acknowledging the reciprocal influence of scholastic achievements and acquired behaviors. The independent variables, Participation in Sports and Physical Activities (PSPA), Involvement in Sports Teams and Clubs (ISTC), and Engagement in Sports Activities (ESA), find support in the ‘Achievement Goal Theory’ and the ‘Social Identity Theory.’ These theories propose that individuals engage in sports to achieve specific goals, be they mastery or performance-oriented, and that group participation in sports teams may shape social identity and influence behavior. Thus, these variables aim to gauge the immediate impact of sports participation and potential social and identity-related aspects associated with different forms of sports involvement.

Incorporating moderating-mediating factors, specifically Cultural Context (CC) and its interactions with SD, CF, and PWB, is grounded in the ‘Cultural-Historical Activity Theory.’ This concept underscores the significance of cultural context in shaping individuals’ behaviors and development within a particular sociocultural milieu. Within the Chinese context, these variables seek to explore how cultural elements may influence or mediate the observed outcomes, providing a nuanced understanding of the interplay between sports participation and educational progress. Finally, the mediating factors such as Perceived Social Development Through Sports (PSDTS), Sports-Induced Cognitive Focus (SICF), and Psychological Well-Being (PWB) align with the ‘Self-Determination Theory’ and the ‘Positive Youth Development Framework.’ These theories posit that sports foster autonomy, competence, and connection, leading to positive social and cognitive outcomes. Examining potential mediators in Psychological Well-Being, such as stress alleviation, self-worth, and adaptability, aligns with the broader recognition that these psychological aspects contribute to shaping one’s overall well-being.

In educational contexts, sports extend beyond mere physical activity, providing advantages for physical well-being, mental resilience, and social skills development [4]. Higher education institutions acknowledge the role of sports in facilitating holistic student development, a significance accentuated in China’s challenging and stress-laden academic environment [5]. This study explores the influence of sports on academic advancement, social maturation, mindfulness, and mental health among Chinese university students.This research carries significance for multiple reasons:

- China’s education system is renowned for its high-pressure environment, often leading to stress and mental health concerns [6].
- China actively advocates for sports in institutions to promote physical and character development, and our study sheds light on the effectiveness of these policies [7].
- The interconnectedness of academic progress, social development, mindfulness, and mental well-being implies that changes in one sphere can ripple across others. This understanding guides institutions in providing holistic student support [8].

The findings hold implications for educators, policymakers, and students.

Educators glean insights on how sports enhance academic outcomes and well-being, guiding policymakers in refining physical education programs. Students may gain motivation for physical activities to manage university challenges better. This understanding is crucial in China, where well-being intersects with academic demands. This research informs policies, aids universities in enhancing support systems, and empowers students to leverage sports for academic and personal development.

This research addresses gaps in existing studies on the impact of sports, specifically in China’s cultural and educational setting. It comprehensively examines the effects of sports on Chinese university students, including academic progress, social development, mindfulness, and mental health. By exploring mediating factors, the study aims to offer a nuanced understanding. It contributes to evidence-based strategies for educational institutions and policymakers in China, prioritizing a holistic approach to student well-being. Overall, this research informs interventions and support systems to enhance students’ well-being and success in Chinese universities.

### Review of literature

The intersection of sports, learning, social progress, meditation, and psychological well-being is globally gaining academic and practical importance. This literature review examines research on the effects of sports on students, especially Chinese university students.

#### Effects of sports on perceived educational progress

Sports’ impact on perceived educational success reveals a complex link between physical exercise and academic performance. While early studies hinted at potential clashes, recent research suggests a mutually beneficial relationship [9, 10]. Sports contribute to physical health, cognition, discipline, and time management. Regular exercise enhances attention, memory, and problem-solving—which is essential for academic success [11]. Team sports cultivate leadership, communication, and collaboration skills, applicable to academics, and offer stress relief in China’s competitive learning environment [12].

Lin et al. (2023) found that chiropractic therapy in China and Hong Kong enhances health and sports performance by addressing biomechanical issues and improving neuromuscular function [13]. The study suggests that increased exposure to global audiences and investors may contribute to the growth of the chiropractic business. Zuo et al. (2023) investigated the interaction between traditional sports and games (TSGs) and nature, revealing that survival values, beliefs, attitudes, and the environment significantly shape TSGs [14]. This research challenges the notion that TSGs are solely influenced by their natural environment, providing theoretical and practical advice for their conservation. Zou et al. (2023) explored factors influencing the continued viewership of Chinese sports fans, finding that perceived value, functional quality, simplicity of use, and service quality impact audience loyalty. The report suggests government, business, and platform solutions to address challenges in audience loyalty. Xu et al. (2023) studied the social media attitudes of prospective Chinese PE teachers, with qualitative analysis revealing insights into value, risk, and overall perspective. Understanding how future educators perceive social media is crucial for its effective use in education [15].

In China, which values academic success, the influence of sports on education is enormous [16]. The nation’s education system is known for its high standards and academic concentration [17]. This paradigm suggests that sports may correct imbalances. This engagement lets kids exercise, improve their cognition, and manage their time.

#### Effects of sports on social development

University students’ entire development depends on social skills. Sports encourage socialization, teamwork, and personal development [18]. Sports teams and clubs help students build leadership, conflict resolution, and interpersonal skills. These skills are valuable in academic and personal/professional settings, according to Foley et al. (2022). In China, where solidarity and harmony are valued, athletics may help students cooperate and adapt to different social situations, according to Gu &Xue et al. (2022). The relationship between sports and social development in Chinese universities may reveal how sports might promote social competence [7, 19].

Jacobo&Calabuig (2022) investigated the perception of athletic events as tourism attractions in Gran Canaria, finding positive effects on social cohesiveness, local growth, and overall sentiment [20]. This research informs tourism strategies and policies. Ma &Kurscheidt (2022) compared the commercialization of Chinese and Western professional football, emphasizing the need to understand the policy context for effective analysis [21]. Zhang and He (2022) explored the role of stress, autonomy, and burnout of Chinese social workers, revealing that increased discretion at work reduces burnout [22]. Their findings suggest ways for social care organizations to manage worker burnout effectively. Luo & Chen (2023) analyzed China’s green energy growth and sports industry concentration, indicating that sports industry concentration may positively influence green energy development [23]. The study concludes with policy recommendations for China’s renewable energy sector.

The relationship between athletics and social development is complicated, and understanding it might have significant implications. Academic competition might make it hard for Chinese university students to form social relationships [24]. Sports may help youngsters make friends, feel included, and build social skills [25].

#### Effects of sports on psychological well-being

The connection between athletics and mindfulness, particularly in Chinese institutions, warrants more research for a comprehensive understanding [26]. Zhang et al. (2023) studied mindfulness in Chinese professional athletes, revealing that its long-term effects on burnout are mediated by sensory avoidance and cognitive fusion, suggesting mindfulness training as a potential solution [6]. Wang et al. (2023) conducted quasi-experimental research on Macau college male basketball players, indicating that 7-week mindfulness training improves attention, concentration, and shooting accuracy [27]. Chang et al. (2023) introduced Mindfulness-based Peak Performance (MBPP), a randomized controlled trial exploring the impact of mindfulness-based interventions on peak athletic performance under stress, expecting to reveal the benefits of mindfulness training for athletes [28].

Luo et al. (2023) examined how mindfulness moderated the connection between bullying and sleep disturbance in Chinese youngsters [29]. Their study suggests that boys who practice mindfulness have fewer sleep issues due to bullying. This study suggests mindfulness may reduce bullying’s effects on children’s sleep. Birrer et al. (2023) examined sports acceptance and mindfulness bibliometric literature from 1969 to 2021 [30]. Previous initiatives, pertinent literature, countries, organizations, magazines, and more are included. Since 2014, performance, flow, and acceptability studies have exploded. Topics, including impact mechanisms, self-compassion, and well-being, indicate a growing interest in the neuroscientific aspects of mindfulness in sports.Chinese university students face tremendous academic pressure; therefore, studying the association between sports engagement and mindfulness might help them manage stress and improve mental health [31].

#### Effects of sports on cognitive focus

Cognitive focus concerns among university students are rising globally, including in China. Academic pressure and social expectations cause stress, anxiety, and depression [32]. Regular exercise releases mood-lifting endorphins and lowers cortisol. Sports can boost self-esteem, social support, and success, which may safeguard mental health.Given China’s rapidly aging population, Wong et al. (2023) recommended healthy and active aging. Their literature review covers the effects of exercise on Chinese people’s physical and mental health during the last 15 years [33]. This report says exercise improves physical, mental, and cognitive health. New ways to encourage the elderly to exercise are also discussed. Early trauma damaged Chinese college students’ mental health years later [34]. Their results suggest that childhood suffering does not predict adult mental health. However, high-intensity exercise mitigates early adversity’s long-term mental health impacts better than lower-intensity exercise. This research suggests that exercise may improve the mental health of college students who have experienced early trauma.

Ju et al. (2023) examined Chinese mental health and air pollution. Fixed effects models with instrumental factors confirm that PM2.5 and ground surface ozone worsen mental health [35]. Research shows that air pollution harms mental health, although regular exercise may mitigate this. This research found that regular exercise may reduce the mental health effects of air pollution. Cao and Liu (2023) evaluated how education, sports, Internet usage, TV, and sleep length affect Chinese teenagers’ academic performance [36]. Intellectual interests, physical activity, and relaxation have been linked to academic success. Conversely, excessive internet and TV use might hinder academic achievement. Furthermore, the role of sad symptoms as a mediator is confirmed. This research highlights the necessity to examine teenagers’ temporal allocation patterns and cognitive capacities.It is essential to understand how sports might improve mental health in China, where societal stigma prevents people from getting help. Chinese universities are more aware of the need for mental health care, and sports may help students improve their mental health.

### Interconnections between sports and society

The research on sports and social development finds several connected results. Team sports constantly foster cooperative problem-solving. These exercises teach collaboration and cooperation via group accomplishment. No environment is better for developing practical communication skills than team sports [37]. Sports help people improve their verbal and nonverbal communication skills and social competence. Stress management is another benefit of athletics [38]. Regular exercise reduces stress and improves mental health. Sports lessons assist people in managing stress in daily life. Leadership and responsibility are developed via sports. Sports teams depend on leadership, whether from captains or players. These experiences provide leadership and responsibility abilities that may be used in numerous life and professional circumstances [39].

Sports boost self-confidence and dispute resolution. Participating in competitive and collaborative sports helps people resolve conflicts. Thus, individuals feel more equipped to tackle personal and professional obstacles [40]. Sports foster lasting friendships and other social bonds. Sports also foster diversity and inclusion—sports help to keep active and healthy [41]. Psychological and emotional advantages are as substantial as physical ones [42]. Sports require setting and attaining objectives, which affects other areas of life. Athletic goal-setting develops self-discipline, resilience, and drive that apply to life [43].

### Theoretical background and research hypotheses

This study explores the complex connection between sports participation and the overall development of Chinese university students. It employs moderatedmediation frameworks to investigate how sports impact educational progress, social growth, mindfulness, and mental health. These frameworks consider that the relationship between sports participation and educational progress can be influenced by mediating factors (intermediate factors explaining the relationship) and moderating factors (contextual factors affecting the relationship’s strength or direction).

#### H1

Participation in sports and physical activities positively correlates with educational progress with mediating factors of social development, cognitive focus, and psychological well-being, while moderated mediation with Chinese culture.

Academic self-efficacy, a mediator, is essential to interpreting the association between sports engagement and academic achievement. Sports may boost students’ self-confidence, which improves their study habits and performance. Further, academic competitiveness and pressure in China may decrease. Sports may relieve stress and enhance time management, benefit academic development more in high-pressure workplaces.

#### H2

Involvement in sports teams and clubs has positively impacted social development, cognitive focus, and psychological well-being, ultimately contributing to improved educational achievement.

Team cohesion, a mediating element, may explain how sports participation boosts social development. Sports teams foster camaraderie and teamwork, which may affect students’ social connections outside of sports. Additionally, Chinese collectivism regulates. Sports may significantly impact social development in collectivist cultures that value group cohesiveness and cooperation.

#### H3

Engagement in sports activities has been shown to correlate positively with enhanced cognitive focus and psychological well-being.

**Fig. 1:**
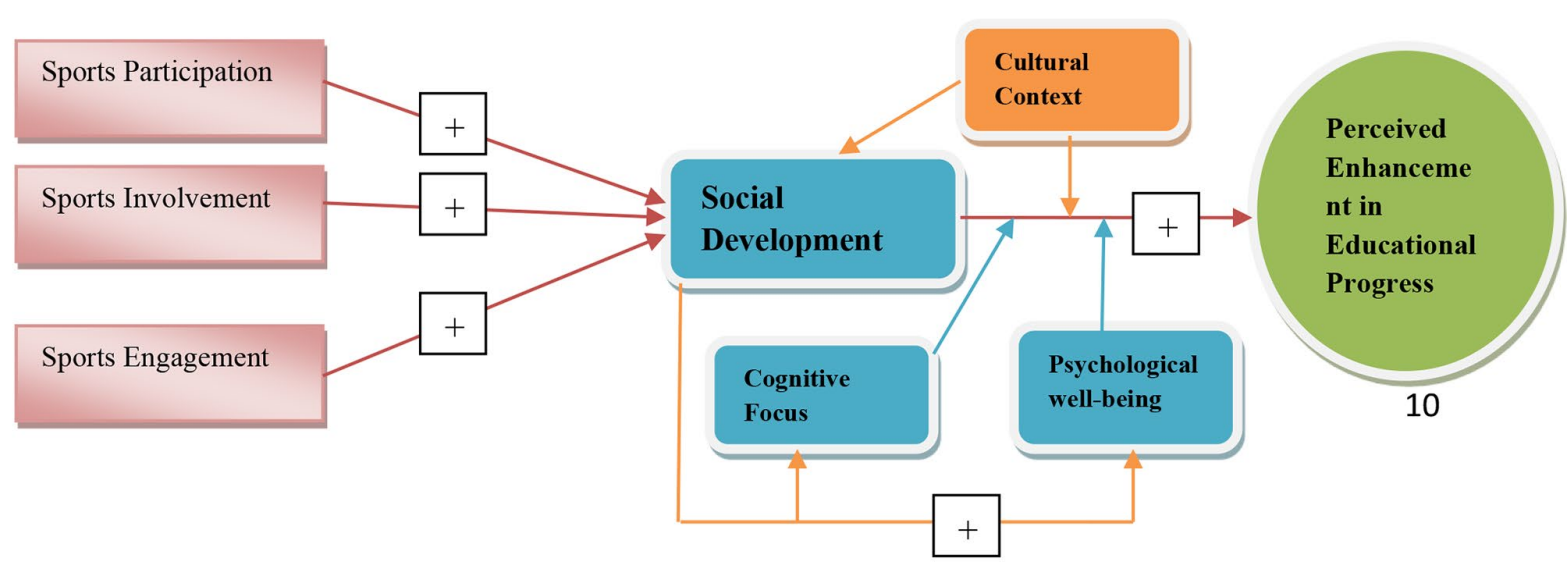
Theoretical framework

This hypothesis suggests that stress reduction, self-esteem, and resilience mediate sports engagement, cognitive attention, and psychological well-being. Sports may reduce academic stress and improve mental health. Additionally, academic stress and mental health support programs may moderate the effect. Athletic activities may improve cognitive attention and psychological well-being in high-pressure academic contexts when students struggle to maintain their mental health. Figure 1 shows the investigation’s theoretical backdrop.

Our moderated mediation framework provides a solid theoretical foundation for studying how sports involvement affects academic advancement, social development, cognitive focus, and psychological well-being in Chinese university students. We hope to grasp these complex connections by studying mediating and regulating elements. We can better understand how sports affect college students’ overall development in China by understanding their theoretical foundations.

### Objectives of the study

This study has three objectives. The first objective is to the impact of sports on the academic progress of Chinese university students. The second objective is to investigate the relationship between sports participation and the development of social skills among Chinese university students. The third objective is to examine the links between Chinese university students’ sports involvement, cognitive focus, and psychological well-being. Additionally, it explores potential relationships between cultural factors and social development factors Furthermore, to investigate the impact of sports on Chinese university students, we address the following research questions:

1. How does engagement in sports influence the academic progress of Chinese university students, encompassing academic achievements and study habits?
2. What role do sports play in the social development of Chinese university students, encompassing interpersonal relationships, teamwork, and leadership skills?
3. How does participation in athletic activities relate to mindfulness and mental health in Chinese university students, and what processes underlie these connections?

## Research method

### Participants

The study focused on university students currently enrolled in various academic institutions across China, encompassing a diverse range of universities. The research aimed to capture the breadth of university students, considering various academic backgrounds, programs, and fields of study to comprehensively represent the student body across Chinese universities. Therefore, a sample of 500 students from prominent institutions such as Peking University, Tsinghua University, Fudan University, Zhejiang University, Nanjing University, Shanghai Jiaotong University, Sun Yat-sen University, Sichuan University, Wuhan University, and Harbin Institute of Technology was selected. To ensure academic diversity, students from different departments and study backgrounds participated in the study. The research utilized a convenient sample method to effectively navigate the dynamic landscape of China’s educational system, ensuring representation from diverse academic institutions and departments. Employing University-Based Stratification, the study categorized the target population into strata using various institutions, allowing for the identification of unique attributes among different academic establishments. Stratum-Level Randomized Filtering was then employed to randomly select participants from each institution stratum, ensuring equal opportunities for all eligible students to participate. This approach aimed to minimize bias and achieve a representative sample, including individuals with varied academic interests and institutional affiliations. The robust Response Rate of 82.6%, with 413 out of 500 distributed surveys completed, enhances the study’s reliability and validity, bolstering the credibility of the research findings.For details on the sample’s response to the questionnaire dissemination through social media and field surveys, refer to Table 2.

**Table 2:** Questionnaire distributions, sample size, and response rate

Universities	Questionnaire Distribution	Questionnaire Collection/Sample Size	Response Rate (%)
Peking University	50	40	80
Tsinghua University	50	41	82
Fudan University	50	45	90
Zhejiang University	50	42	84
Nanjing University	50	41	82
Shanghai Jiao Tong University	50	38	76
Sun Yat-sen University	50	43	86
Sichuan University	50	42	84
Wuhan University	50	41	82
Harbin Institute of Technology	50	40	80
Total	500	413	82.6

Source: Field survey

Table 3 shows 413 research participants. The research included 232 males (56.2%) and 181 women (43.8%). Most individuals were young adults (18–25), 32% were adults (26–33), and 10% were seniors (34+). Regarding the enrolment of students, 289 individuals, constituting 70.0% of the participants, were identified as undergraduate students. Conversely, 124 participants, accounting for 30.0% of the sample, were classified as graduate students. Moreover, the health condition of the individuals in our sample was shown to be a differentiating factor. Respondents were queried, ‘Do you engage in any sports or physical activities as a student?’ - Affirmative / - Negative. The participants who answered ‘Yes’ were classed as student-athletes, while those who answered ‘No’ were categorized as non-athletes. The binary categorization was determined by the respondents’ self-reported involvement status, enabling a definitive differentiation between persons actively involved in sports or physical activities and those not.Out of the total sample size, 261 participants (63.2%) were classified as student-athletes, while the remaining 152 participants (36.8%) were categorized as non-athletes. The analysis of the participants’ academic achievement, as assessed by their Cumulative Grade Point Average (CGPA), indicated that 268 participants (64.9%) obtained a CGPA of 3 or less, and 145 individuals (35.1%) attained a CGPA higher than 3. The sample above characteristics provide useful insights into the demographic makeup of our study population. These characteristics will serve as a fundamental basis for the forthcoming analyses, which will investigate the impact of sports on several aspects of learners’ scholastic achievement, social development, mindfulness, and mental health within the context of China. Table 3 shows the demographic profile of the participants.

**Table 3:** Demographic survey

Sample Characteristics	Category	Frequency	%
Gender	Male	232	56.2
	Female	181	43.8
Age	> 18 to 25 years	238	57.6
	26 to 33 years	132	32.0
	> 33 years	43	10.4
Student’s Enrolment	Undergraduate Students	289	70.0
	Graduate Students	124	30.0
Health	Student-Athlete	261	63.2
	Non-Athlete	152	36.8
CGPA	Up to 3CGPA	268	64.9
	> 3CGPA	145	35.1

Source: Author’s survey

### Instrumentation

Various instruments were employed in this study to measure different constructs. The first instrument utilized was the “Perceived Enhancement in Educational Progress (PEP) measurement,” comprising 5 items adapted from the scholarly works of Spittle & Byrne [44], Crust et al. [45], and Slavinski et al. [46].The second instrument focused on social development, encompassing three factors: Involvement in Sports Teams and Clubs (ISTC), Engagement in Sports Activities (ESA), and Perceived Social Development Through Sports (PSDTS). The 5 items for ISTC and ESA were designed and extracted from the scholarly works of Spittle & Byrne (2009), Curst et al. (2014), and Slavinski et al. (2021) [44–46]. The 5 items for PSDTS were extracted from the scholarly work of Conroy &Coatsworth (2006) and Spaaij (2009) [47–48].

The third instrument focused on Cultural Context (CC), and the items were extracted from the scholarly work of Sue & Zane [49] and Yu et al. [50]. An example question is, “The cultural context in China significantly influences the emphasis placed on sports as part of the education system.”

The fourth instrument, Sports-Induced Cognitive Focus (SICF), drew upon the academic research conducted by Baltzell and Akhtar [51] and Scott-Hamilton et al. [52]. It aimed to identify key elements of SICF, focusing on “The positive impact of participating in sports activities on an individual’s ability to cultivate mindfulness and enhance awareness of the present moment.”

The fifth instrument, Psychological Well-Being (PWB), comprised 5 items derived from the academic research conducted by Mahoney et al. (2014) and Breistøl et al. (2017) [53–54]. A sample question is, “Participating in sports activities benefits one’s holistic mental well-being.”

### Data collection procedure

#### Data collection

Data collection spanned from February 15th to June 25th, 2023, followed by the data analysis phase until July 30th, 2023, strategically chosen to encompass all pertinent research variables and conduct a thorough examination of the collected data. Employing a dual approach for data collection, the study utilized popular social media platforms (WeChat, Weibo, and QQ) and structured questionnaires, effectively communicating with potential students and encouraging their questionnaire participation. The questionnaires were administered to students at their respective universities, preceded by a pilot test with a smaller student group to ensure item clarity and comprehension. The study assessed the reliability of scales using Cronbach’s alpha to ensure internal consistency.

In the subsequent analysis, the study considered various variables, including the dependent variable Perceived Educational Progress (PEP), which encompassed academic performance and study habits. Independent variables comprised Participation in Sports and Physical Activities (PSPA), Involvement in Sports Teams and Clubs (ISTC), and Engagement in Sports Activities (ESA). The study introduced mediating variables, such as Perceived Social Development Through Sports (PSDTS), Sports-Induced Cognitive Focus (SICF), and Psychological Well-Being (PWB), potentially influenced by factors like stress reduction, self-esteem, and resilience.

Moreover, the study explored moderating-mediating variables, focusing specifically on Cultural Context (CC) and its interactions, including CC × SD, CC × CF, and CC × PWB. This exploration delved into China’s cultural and educational context as a critical element in the overall analysis.

#### Data analysis

The data underwent diverse analytical procedures. Initially, descriptive statistics, encompassing means, standard deviations, and frequencies, were computed to offer a comprehensive overview of the sample and the variables under investigation.Subsequently, Cronbach’s Alpha was employed as the second analytical step to evaluate the reliability of the scales utilized in the study. This measure aimed to ensure robust internal consistency for each scale, thereby bolstering the reliability of the measurement instruments.As the third analytical approach, factor analysis, a statistical technique, was applied. This method sought to investigate the latent component structure of the observed data, unveiling underlying factors contributing to the observed patterns.

Finally, the fourth method involved the utilization of regression analysis to empirically examine relationships between deterministic, reliant, controlling, and intervening factors. This approach facilitated hypothesis testing and provided a deeper understanding of the intricate relationships between various variables.

## Results

This study shows how sports affect Chinese students’ academic progress, social growth, awareness, and psychological well-being. The study provides valuable information about sample characteristics and measuring scale reliability. Table 4 displays the descriptive statistics and Cronbach’s Alpha coefficients for the variables examined in our research, which investigates the influence of sports on scholastic advancement, social growth, cognitive focus, and psychological well-being in China.

**Table 4:** Descriptive statistics and Cronbach’s Alpha

Variables	N	Range	Mean	Std. Deviation	Variance	Cronbach’s Alpha
Gender	413	1	0.561	0.496	0.247	-----
Age	413	2	1.527	0.677	0.459	-----
Enrolment	413	2	1.302	0.465	0.216	-----
Health	413	1	1.368	0.482	0.233	-----
CGPA	413	1	1.351	0.477	0.228	-----
PEP	413	3	3.682	0.534	0.286	0.741
PSPA	413	3	3.647	0.552	0.306	0.792
ISTC	413	3	3.688	0.569	0.324	0.851
ESA	413	3	3.678	0.569	0.325	0.874
CC	413	3	3.659	0.592	0.351	0.910
PSDTS	413	3	3.663	0.566	0.321	0.771
SICF	413	3	3.674	0.582	0.339	0.749
PWB	413	3	3.659	0.573	0.329	0.801

Source: Author’s estimate

Table 4 outlines the key characteristics of our sample. The gender distribution tilts towards males (56.2%) compared to females (43.8%), a factor essential for analyzing the impact of sports on education and psychology. The average age, 1.527, implies that the majority fall within the 18–33 age range, offering insights into how different age groups respond to sports-based learning. Student enrollment statistics reveal 70.0% undergraduates and 30.0% graduates, suggesting the need for varied sports program designs based on student demographics. Non-athletes (36.8%) and student-athletes (63.2%) exhibit distinct health profiles, allowing for an assessment of the mental and educational benefits of sports. Notably, 64.9% of the sample holds a CGPA of three or below, providing a basis for comparing academic performance with sports activity.

The specific criteria of interest highlight that participants’ perceived educational progress (PEP) averages 3.682, indicating positive educational advancement. Sports and physical activities (PSPA) garner a slightly lower mean score of 3.647, signifying moderate participation. Considerable engagement in team-based sports at ISTC is reflected in a mean score of 3.688. ESA achieves a typical score of 3.678, indicating robust sports activity. Cultural context (CC), representing China’s cultural and educational background, averages 3.659, suggesting cultural influences on individuals. Perceived Social Development Through Sports (PSDTS), encompassing cooperation, leadership, and interpersonal relationships, earns a mean score of 3.663, indicating a favorable environment. The average SICF is 3.674, denoting moderate mindfulness. Psychological well-being (PWB), covering stress reduction, self-esteem, and resilience, averages 3.659, reflecting cognitive positivity.

Individual variable Cronbach’s Alpha values indicate measuring scale internal consistency. Data credibility increases with higher Cronbach’s Alpha values (0.741 to 0.910), indicating scale reliability. Significant component variance and total variance are in Table 5.

**Table 5:** Principal component analysis

Component	Initial Eigenvalues			Extraction Sums of Squared Loadings		
	Total	% of Variance	Cumulative %	Total	% of Variance	Cumulative %
1	1.272	15.898	15.898	1.272	15.898	15.898
2	1.116	13.950	29.848	1.116	13.950	29.848
3	1.094	13.675	43.523	1.094	13.675	43.523
4	1.063	13.293	56.816	1.063	13.293	56.816
5	0.949	11.864	68.680			
6	0.923	11.535	80.215			
7	0.827	10.332	90.547			
8	0.756	9.453	100			

Source: Author’s estimate

Table 5 reveals that the first component, with an initial eigenvalue of 1.272, explains 15.898% of the variation. The second component, with an initial eigenvalue of 1.116, accounts for 13.950% of the variance. Combined, the first two components explain 29.848% of the variation. The third component, with an initial eigenvalue of 1.094, explains 13.675% of the variation. The first three components together represent 43.523% of the variance. The fourth component, with an initial eigenvalue of 1.063, explains 13.293% of the variance. The first four components collectively capture 56.816% of the variation. Components 5 to 8, although having eigenvalues, are not analyzed or presented in the table as they fall below the threshold for meaningful interpretation (eigenvalue < 1). Principal Component Analysis (PCA) was conducted using the “Extraction Method,” demonstrating that the top four components retain significant information from the original variables, making them meaningful for further analysis. Future studies may disregard components 5 to 8 due to their minimal explanatory power.

Table 6 shows the Factor Analysis findings for our research variables. This table’s “Component Matrix” displays the connections between original variables and extracted factors (components).

**Table 6:** Factor analysis

Component Matrix^a^				
	Component			
	1	2	3	4
PEP	0.021	0.058	0.441	0.687
PSPA	0.669	0.112	0.056	0.276
ISTC	0.481	− 0.541	− 0.169	0.139
ESA	0.273	0.252	0.327	− 0.553
CC	− 0.521	− 0.263	0.509	0.186
PSDTS	− 0.265	0.068	− 0.706	0.298
SICF	0.337	0.481	0.026	0.243
PWB	− 0.250	0.663	− 0.055	0.083
a. 4 components extracted.				

Source: Author’s estimate

The Factor Analysis results reveal significant associations between identified components and key variables. Component 1 positively correlates with Perceived Enhancement in Educational Progress (PEP) and Participation in Sports and Physical Activities (PSPA), indicating that integrating sports into educational programs may enhance student academic performance. Component 2, linked to Involvement in Sports Teams and Clubs (ISTC), Sports-Induced Cognitive Focus (SICF), and Psychological Well-Being (PWB), suggests that sports team engagement may not necessarily improve mindfulness or mental wellness. Component 3 is positively connected to Perceived Social Development Through Sports (PSDTS) and Sports Activities (ESA), suggesting that sports contribute positively to social development, offering an avenue for enhancing students’ social, teamwork, and leadership skills. Lastly, Component 4 exhibits favorable associations with Cultural Context (CC) and Sports-Induced Cognitive Focus (SICF), indicating that Chinese educational and cultural variables may influence mindfulness. These findings provide valuable insights for educational institutions to design holistic programs that effectively integrate sports, education, mindfulness, mental health, and social development elements.

A regression analysis is shown in Table 7 to determine how demographic, explanatory, mediatory, and moderating factors affect “Perceived Educational Progress.” Also examined is the moderated-mediation effect.

**Table 7:** Regression analysis

Variables	Demographic Effect	Explanatory Effects	Mediating Effects	Moderating Effect	Moderated-Mediating effect
Constant	3.726***	3.523***	3.529***	3.420***	3.523***
Gender	0.070	-----	-----	-----	-----
Age	− 0.066***	-----	-----	-----	-----
Enrolment	− 0.029	-----	-----	-----	-----
Health	− 0.047	-----	-----	-----	-----
CGPA	0.056**	-----	-----	-----	-----
PSPA	-----	0.055**	-----	-----	-----
ISTC	-----	0.012***	-----	-----	-----
ESA	-----	− 0.016	-----	-----	-----
PSDTS	-----	-----	− 0.007	-----	− 0.625**
SICF	-----	-----	0.042*	-----	0.050
PWB	-----	-----	0.034**	-----	0.557**
CC	-----	-----	-----	− 0.314	
CC*PSDTS	-----	-----	-----	0.963***	0.887**
CC*SICF	-----	-----	-----	0.203**	− 0.024
CC*PWB	-----	-----	-----	− 0.606	− 0.777**
**Statistical Test**					
R^2^	0.312	0.412	0.389	0.401	0.547
Adjusted R^2^	0.298	0.387	0.341	0.356	0.501
F-statistics	4.525***	5.784***	4.835***	4.986***	6.012***

Source: Author’s estimate. Note: Dependent variable: PEP. ***, **, and * shows 99%, 95%, and 90% significance level

Educational institutions face significant economic and administrative challenges when “Age” lowers students’ performance. To help older students thrive, they need financial aid, counselling, and academic support [55]. This investment may improve employment chances and incomes, benefitting society. Institutions administrators should provide older students with specialized academic assistance, career coaching, and flexible class schedules to fit their employment and family obligations [56]. Meeting older kids’ needs improves their education and performance.

Higher CGPAs improve educational results, making it essential. For education and economic prosperity, institutions should emphasize intellectual achievement. Academically challenging institutes create graduates with suitable skills and knowledge, improving their career chances and income [57]. Institutions should give resources and subsidies to motivate students to obtain high CGPAs. Tutoring, study groups, and academic help may boost academic performance [58]. Recognizing CGPA’s relevance may help institutions set clear academic expectations and provide students with the tools and assistance they need to excel.

“PSPA” improves academic performance, showing that sports and physical exercise boost academic performance. Educational institutions face economic and management repercussions from this conclusion. Institutions should prioritize and support sports and physical activity programs to strategically improve health and education [59]. Diverse sports, physical activities, infrastructure, coaching, and extracurricular teams may boost student participation. The curriculum may also include sports-related themes and concepts to promote transdisciplinary learning [60]. Support systems and mentorship programs that relate academic performance to sports engagement may further reinforce the bond.

The positive influence of “ISTC” on educational advancement underlines the relationship between extracurricular sports and academic performance. Extracurricular activities improve academic achievement, graduation rates, and workforce skills [61]. Institutions should invest in and develop sports teams and organizations for better education and campus life. These initiatives need coaching, facilities, and equipment [62]. Institutes may use sports teamwork, leadership, and discipline to teach life skills. Mentorship programs that link sports and academics may help pupils. Extracurricular activities like sports teams and organizations may improve learning [63].

The link between SICF and academic achievement emphasizes the necessity of cognitive methods to improve student performance. Student awareness improves graduation rates, academic performance, and workforce competency, boosting economic development [64]. Meditation programs, mindfulness training, and seminars may be added to educational programs and student assistance. These approaches assist learners in managing stress, increasing focus and general well-being, and improving academic achievement. Mindfulness training and incorporation into teaching may boost classroom engagement, focus, and emotional self-regulation [65]. These treatments should be part of a balanced student well-being and academic performance plan.

Mental health affects academic performance. Thus, institutions should include mental health treatments in achievement programs. Mentally healthy students graduate faster, perform better, and work more efficiently, which boosts economic development and social welfare [66]. Student accomplishment programs should include counselling, stress management, mental health specialists, and stigma reduction measures. A caring and inclusive campus environment that promotes mental health awareness and encourages students to seek help is essential. Public education, peer support, and mental health services may address mental health difficulties [67]. Regular assessments of pupils’ mental health and well-being may identify individuals who need support and adapt remedies. Recognizing the link between mental health and academic achievement helps institutions aid students emotionally and intellectually.

PSDTS has a negative mediation impact on Cultural Context (CC) and educational development, meaning social progress mitigates the detrimental effects of cultural background on schooling. From a management standpoint, social development skills must be improved to offset cultural influences on educational advancement. Social development determines how cultural background affects institutions [68]. CC and PSDTS moderate and moderated-mediates education. This complicated link emphasizes adjusting education to students’ cultural and social development. To address educational disparities across cultures and socioeconomic groups, institutional strategies should incorporate cultural context and socioeconomic development determinants to provide equitable access to educational resources and opportunities [69]. Proactive support programs concentrating on culturally sensitive teaching, mentoring, and interventions to meet different students’ needs may benefit educational institutions [70]. Data-driven decision-making may assist in building student-support strategies and approaches by understanding how cultural background and social development impact educational attainment. Teachers need more training to understand cultural context and social evolution and create inclusive, culturally sensitive learning settings [71].

Cultural context matters for introducing mindfulness programs in institutions since CC and SICF moderate educational attainment favourably. Culturally customized mindfulness practices may improve educational development and student performance, boosting the economy [72]. Educational institutions should provide management-level mindfulness programs representing students’ cultural backgrounds. Faculty and staff need cultural competence training to comprehend and use cultural context [73]. This program helps teachers develop inclusive and culturally sensitive classrooms.

Cultural context and mental health may impair educational growth, as CC and PWB (Psychological well-being) adversely influence academic advancement. This shows how culturally responsive mental health treatment improves learning and long-term economics. Institutions should include counselling, stress management, and mental health awareness programs that integrate students’ cultures. These programs should address culturally diverse students’ concerns and fight mental health stigma [74]. To ensure students obtain mental health care, collaboration with culturally relevant treatment providers is essential [75].

## Discussion

Upon analyzing the empirical outcomes of our research, a complex network of components becomes apparent, each exerting a noticeable impact on the learning setting. By using well-established theoretical frameworks, we want to carefully analyze these subtle distinctions and provide an academic discussion on the consequences for higher learning. The connection between physical activity (PSPA) and academic achievements becomes apparent when considering the alignment with embodied ‘cognitive theory.’ The embodiment viewpoint suggests that cognition is intrinsically connected to sensory experiences. In line with Davids et al. [76] influential research, our results support the need to reassess instructional goals. Institutions should prioritize and support physical endeavours and sports to enhance both academic performance and students’ general health, considering the connection between the body and the mind. The apparent inverse relationship between age and academic success prompts us to consider the ‘socioemotional selectivity theory.’ According to Pruzan&Isaacowitz [77], this idea emphasizes how people’s priorities change as they age, with a greater focus on emotionally significant objectives. To conform with this framework, institutions should readjust their support systems to cater to the distinct requirements of adult learners. The integration of financial assistance, counselling, and learning support is necessary for the school and a moral obligation.

The work supports the principles of ‘human capital theory’ and confirms the inherent worth of cognitive accomplishment, as shown by higher CGPAs. The notion of talent, advocated by Pang & Lee [78], explains the significant influence that learning has on economic output. The institution’s focus on academic rigor, bolstered by resources, scholarships, and mentoring programs, fosters a mutually beneficial connection between intellectual endeavour and economic success. The fundamental relationship of SICF highlights the significance of ‘cognitive learning theories.’ The research highlights the need to incorporate meditation programs and mindfulness therapies into the instruction system, promoting a shift toward comprehensive intellectual growth. Grounded on the ‘biopsychosocial paradigm,’ the connection between emotional wellness and academic success emphasizes the complex interaction of psychological and social variables.

The research focuses on the favorable effects of recreational pursuits on academic attainment, keeping with the youth growth vision. Wakefield & Poland [79]emphasize the significance of social events in promoting proficiency, moral qualities, and social bonds. Our research suggests that educational institutions should invest significantly in various sports programs. These programs should go beyond just providing recreational activities and instead serve as catalysts for the whole development of students. The presence of PSDTS in the relationship between Cultural Context (CC) and learning growth leads us to explore the concept of ‘social capital theory’. Pusztai [80] proposes that interpersonal relationships and shared resources influence educational achievement according to this paradigm. The research suggests that institutions should prioritize acquiring interpersonal capabilities to mitigate the influence of cultural origins on educational progress. Incorporating inclusive methods of instruction and proactive assistance programs is essential in the quest for instructional justice. The research highlights the need for learning institutions to include integrated psychological interventions in student success programs. Institutions may enhance their students’ intellectual and psychological well-being by creating a supportive campus atmosphere. This will not only lead to academic achievement but also help in developing resilient and flourishing persons.

The intersection of CC and PWB in influencing academic progress leads us to behavioural neuroscience. Emphasized by James &Prilleltensky [81] research, cultural psychology highlights the mutual impact of civilization and emotional wellness. Our research emphasizes the need for learning organizations to include indigenous behavioural healthcare therapies in educational support programs. Engaging in partnerships with culturally appropriate treatment providers is crucial to effectively address the complex psychological medical requirements of various student groups. The influence of Cultural Context (CC) on implementing mindfulness programs requires examining cultural competency frameworks. These frameworks, influenced by Dyche& Zayas [82], emphasize the need to understand and integrate cultural subtleties into educational processes. The research strongly recommends that institutions of learning go beyond standard approaches and instead promote incorporating mindfulness practices tailored to specific cultural contexts. An aware and knowledgeable faculty, proficient in cultural competency, plays a crucial role in establishing diverse and socially aware environments.

## Conclusions and policy recommendations

This study examines how Chinese university students’ sports participation affects their academic performance, social development, cognitive concentration, psychological well-being, and cultural environment. To study these factors’ complicated interplay, 413 students from 10 Chinese institutions were surveyed. The results illuminate several academic progression elements. Age seems to affect educational attainment negatively. However, a positive association between CGPA and educational progress demonstrates that academic achievement leads to educational advancement. Sports and physical activities (PSPA) and sports teams and clubs (ISTC) also improve academic performance. According to the findings, sports promote academic performance and social development. Also, Sports-Induced Cognitive Focus (SICF) and psychological well-being (PWB) improve perceived educational success. This research emphasizes the need for mental health advocacy and mindfulness in institutions. Cognitive attention and psychological well-being offset the unfavourable effects of Perceived Social Development Through Sports (PSDTS) on academic progress. This complex link emphasizes the necessity for a complete educational aid plan that includes mental health and social development. The moderation study showed that the impact of these components differed by culture. The positive relationship between social development and academic performance is boosted by cultural background. However, the connection between cultural environment and psychological well-being had a conflicting effect, suggesting a complicated interaction between culture and mental health in educational development.

The research findings support various comprehensive policy recommendations to promote Chinese university students’ educational quality and well-being, such as,

I. Implementing inclusive student support programs is essential for addressing academic, mental health, and physical fitness aspects of student well-being. These programs should be designed to safeguard pupils at different educational levels.
II. Promoting an interdisciplinary approach to teaching is crucial for integrating sports and physical activities into the curriculum. This method promotes a balanced lifestyle since physical and mental health affect academic performance.
III. Cultural competence training is essential for educators at all levels to provide inclusive and culturally aware learning environments. This training program aims to foster tolerance for diverse viewpoints.
IV. Prioritizing mental health care in educational institutions is crucial. This includes ensuring students access counselling, stress management, and mental health awareness activities. Eliminating the stigma of mental health patients is crucial.
V. Promoting and supporting initiatives to improve physical fitness is essential. This requires vigorously promoting regular sports and exercise. A holistic method enhances students’ physical, mental, and academic well-being.
VI. It is crucial to provide flexible educational options for all age groups. Customized programs should help older students overcome educational hurdles and continue their academic pursuits.
VII. Establishing institutional mechanisms like scholarships, awards, and honours programs is crucial for formalizing academic excellence recognition and incentives. This fosters academic achievement and progress.
VIII. Offering diverse extracurricular activities, including sports teams, organizations, and cultural activities, is significant. These activities promote social growth, leadership, and well-roundedness.
IX. Evaluation of policies and programs’ impact on students’ well-being and academic achievement is crucial. Research-driven policymaking helps make informed changes and improvements.
X. Fostering cooperation across educational institutions, government agencies, non-profits, and corporations is crucial. These collaborations may leverage resources and expertise to help children develop fully.

The policy above promotes a holistic approach to education that addresses student well-being’s physical, mental, and social elements. By implementing these steps, China may create an educational system that promotes academic success and holistic development, preparing students for future challenges.

Our study’s findings have significant pedagogical implications for educational settings. Teaching strategies should be adapted to meet the specific needs of more aging students by providing them with professional support services, including counseling, flexibility, and financial aid. In addition to traditional academic assessments, a holistic approach that encourages analytical thinking, creativity, and problem-solving should be advocated to reevaluate the emphasis on cognitive achievements. Because of the positive relationship between physical activity and academic performance, institutes should attempt to include physical education and sports in their curricula to help students stay healthy and sharp. When looking for ways to help students develop their minds, manage stress, and feel better emotionally, contemplative courses and meditation exercises are great resources. Training teachers and other institute employees to be culturally competent are essential for creating welcoming classrooms that receive students from all backgrounds and perspectives. Achieving academic equity requires proactively launching aid initiatives that address socioeconomic and ethnic issues head-on. Institutions must transform and enhance their methods to create a more inclusive, comprehensive, and supportive learning environment for all students as extracurricular involvement expands beyond traditional boundaries and mental health is integrated into the educational experience.

## Data Availability

Data is provided within the manuscript, and it will available upon request from the corresponding author.
